# KALGAE™ (*Klebsormidium flaccidum* var. ZIVO) dried algal biomass - 90-day dietary toxicity study and genotoxicity studies

**DOI:** 10.1016/j.toxrep.2018.09.002

**Published:** 2018-09-20

**Authors:** Julie A. Brickel, Ray A. Matulka, Amy E. Steffek

**Affiliations:** a Burdock Group, 859 Outer Road Orlando, FL, 32814, USA; b ZIVO Biosciences, Inc., 2804 Orchard Lake Road, Suite 202, Keego Harbor, MI, 48320, USA

**Keywords:** Microalgae, Plant-based protein, Toxicity, Mutagenicity, KALGAE™, *Klebsormidium flaccidum*

## Abstract

Consumers are demanding plant-based alternative protein sources to complement traditional animal-based protein sources to fulfill their dietary protein requirements. KALGAE™, a dried algal biomass derived from *Klebsormidium flaccidum* var. ZIVO, is a potential source of plant-based protein that has been evaluated for safety to support its use as a food ingredient. There were no treatment-related adverse effects observed in the 14-day palatability/toxicity or 90-day dietary toxicity study in CRL Sprague-Dawley CD^®^ IGS rats. In the 90-day study, KALGAE™ was administered to rats in the diet at 0, 37,500, 75,000, or 150,000 ppm. No adverse effects were attributed to the test substance for the following parameters: body weight, body weight gain, mean food consumption and efficiency, hematology, clinical chemistry, urinalysis, gross pathology, histopathology, or organ weights. Although some statistically significant effects were recorded, the effects were not considered to be of toxicological relevance. A No Observable Adverse Effect Level (NOAEL) of 150,000 ppm, equivalent to dietary intakes of 7895.2 (male) and 9708.09 (female) mg KALGAE™/kg body weight/day in rats was established. KALGAE™ was non-mutagenic in the *in vitro* bacterial reverse mutation assay at concentrations up to 5000 μg/plate (with or without S9 metabolic activation), nor was KALGAE™ genotoxic in the *in vivo* mammalian erythrocyte micronucleus test in Swiss albino (ICR) mice. These results support the safe use of KALGAE™ as an ingredient in foods.

## Highlights

• NOAEL of 7895.2 mg/kg bw/day (male) and 9708.09 mg/kg bw/day (female) rats.
• Non-mutagenic in *in vitro* bacterial reverse mutation assay.
• Non-genotoxic in the *in vivo* mammalian erythrocyte micronucleus test.

## 1 Introduction

Consumer demand for alternative protein choices continues to increase, with plant-based protein options reducing the more traditional animal-based protein sources in the diet, such as whey, meat, dairy, and eggs. Consumers perceive plant-based protein alternatives as nutritious, healthy, and sustainable food choices that allow consumers to meet daily nutritional requirements.

Over recent years, alternative protein sources have made their way onto grocery market shelves [1]. ZIVO Biosciences, Inc. (Keego Harbor, MI) (hereinafter referred to as “ZIVO”) has harvested and optimized a strain of green freshwater microalgae derived from *Klebsormidium flaccidum*, which has since been named *K. flaccidum* var. ZIVO. *K. flaccidum* var. ZIVO is sustainably harvested and is a potential source of nutrients with substantial amounts of protein, vitamin K_1_, niacin, and polyphenols. The microalgae are in constant cultivation in open-air, partially open-air, and green-house covered raceway ponds. When ready for harvest, the microalgae are removed from the ponds and the medium is separated into biomass and supernatant. Subsequently, the biomass is rinsed with potable water, dried, and stored in polyethylene, food-safe bags inside of rigid fiber drums at ambient temperature. The dried algal biomass derived from *K. flaccidum* var. ZIVO has been named KALGAE™. Unlike other sources of microalgae that may have an undesirable taste or odor, KALGAE™ is tasteless and nearly odorless, therefore obviating the need for masking agents and contributing to its versatility as a plant-based protein source. ZIVO intends for KALGAE™ to be used as a source of protein and nutrients in food bars; vegetarian soups, stews, and chili; nutritional drinks; nutritional drink mixes; smoothies and juices; snack foods; dried vegetable protein mixtures; powdered, leaf, and bottled teas; and as a condiment in salads and pastas.

To evaluate the safety of KALGAE™ as an ingredient in foods, a 14-day palatability/toxicity study and a 90-day dietary toxicity study were conducted in rats. Also, the genotoxic potential of KALGAE™ was assessed in an *in vitro* bacterial reverse mutation assay and an *in vivo* mammalian erythrocyte micronucleus test.

## 2 Materials and methods

### 2.1 Test substance and diet preparation

KALGAE™ is a dried algal biomass powder derived from *K. flaccidum* var. ZIVO and is brown to green in color. KALGAE™ contains a substantial amount of protein (≥40 g/100 g), vitamin K_1_ (≥150 μg/100 g), niacin (≥1.5 mg/100 g), and polyphenols (≥300 mg/100 g). The product characteristics are provided in Table 1. KALGAE™ was tested for microbial and heavy metal contaminants, and all test results were below the respective limits (Table 1).

**Table 1:** Product characteristics for KALGAE™ (*K. flaccidum* var. ZIVO).

Physical Properties		Microbiological Contaminants		Heavy Metals (ppm)		Nutritional Information (*per* 100 g)	
Appearance	Fine powder	Aerobic Plate Count	≤10,000 CFU/g	Arsenic	≤2.0		
Color	Brown - green	Yeast and Mold	<30 CFU/g	Lead	≤2.0	Fat	≥7%
Moisture	≤15%	*Salmonella*	Negative/25g	Cadmium	≤1.0	Total CHO	≥25%
Ash	≤10%	Total coliforms	<3 MPN/g	Mercury	≤1.0	Dietary Fiber	≥18%
		*Staphylococcus aureus*	<10 CFU/g			Sugars	≤2%
		Enterobacteriaceae	<10 CFU/g			Protein	≥40%
		*Escherichia coli*	<3 MPN/g				

CFU, colony forming units; CHO, carbohydrates; MPN, most probable number; ppm, parts *per* million.

KALGAE™ was also tested periodically for pesticides [2], pheophorbide a (free and bound; HPLC), and the following cyanotoxins (analytical methodology): microcystins (ELISA), cylindrospermopsin (ELISA), saxitoxin (ELISA), anatoxin-a (LC–MS/MS), lyngbyatoxin-a (LC–MS/MS), debromoaplysiatoxin (LC–MS/MS), aplysiatoxin (LC–MS/MS), and β-*N*-methylamino-l-alanine (LC–MS/MS). The pesticides analysis was conducted by Covance Laboratories (Greenfield, IN), and the cyanotoxin testing was conducted by GreenWater Laboratories (Palatka, FL). No pesticides or cyanotoxins were detected above the respective methodology detection limits (data not shown). The free and bound pheophorbide a results were below the limits established by the Japanese Ministry of Public Welfare for existing (0.8 mg pheophorbide/g microalgae) and total (1.2 mg pheophorbide/g microalgae) pheophorbides [3].

#### 2.1.1 14-day palatability/toxicity study and 90-day dietary toxicity study

KALGAE™ was supplied by ZIVO Biosciences, Inc. (Keego Harbor, MI) (14-day study: batch #: PCS Pilot050217; PSL Reference Number 170511-2D; 90-day study: batch #: ZB-068; PSL Reference Number 170817-6D). The Open Standard Diet (OSD) D12072001 (Research Diets, Inc., New Brunswick, NJ) was used to formulate experimental diets with KALGAE™ to achieve the appropriate target doses and ensure a consistent nutrient profile was delivered at each dose. The diets were prepped weekly and refrigerated. The neat test substance and prepared test diets were sampled in duplicate. Prior to study initiation and termination, KALGAE™ (neat) was tested for stability. To assess the stability of KALGAE™ in the diet, the test diet was sampled on Days 1, 4, 7, and 10. The homogeneity of the diet was also assessed in multiple representative samples. For the 90-day study, one sample was taken from the basal diet to assess homogeneity. The concentration of KALGAE™ in the diet was also verified, utilizing *beta*-carotene as a marker compound. The methodology for the testing was suitable and validated.

#### 2.1.2 *In vitro* bacterial reverse mutation assay

KALGAE™ was supplied by ZIVO Biosciences, Inc. (Keego Harbor, MI) (batch #: PCS Pilot050217; PSL Reference Number 170511-2D).

#### 2.1.3 *In vivo* mammalian erythrocyte micronucleus test

KALGAE™ was supplied by ZIVO Biosciences Inc. (Keego Harbor, MI) (batch #: ZB-037; PSL Reference Number 170206-1D). The Harlan Teklad Global 16% Protein Rodent Diet^®^ #2016 was supplied by Envigo Teklad Laboratories (East Millstone, NJ) and KALGAE™ was added to the feed.

### 2.2 Chemicals and materials

For the 14-day palatability/toxicity study and the 90-day dietary toxicity study, filtered tap water was supplied to the animals *ad libitum*. Analyses of water are conducted and recorded on a regular basis (Precision Analytical Services, Inc., Toms River, NJ and South Brunswick Municipal Water Supply, South Brunswick, NJ). The potential for known contaminants in the food or water was minimal and therefore unlikely to impact the outcomes of the studies.

For the bacterial reverse mutation assay, the S9 liver fraction, sourced from male Sprague-Dawley rats induced with phenobarbital and benzoflavone, was purchased from Molecular Toxicology, Inc. (Boone, NC). The positive controls (*i.e.,* sodium azide (NaN_3_), ICR 191 acridine, daunomycin, methyl methanesulfonate (MMS), 2-aminoanthracene (2-AA)), overlay agar (which included biotin, histidine, and tryptophan), and minimal glucose agar plates were obtained from Molecular Toxicology, Inc.

For the *in vivo* mammalian erythrocyte micronucleus test, the positive control substance, cyclophosphamide monohydrate (batch #: MKBX1822 V; PSL Reference Number 160816-11 H), was purchased from Sigma-Aldrich (St. Louis, MO). Corncob-based bedding (bed-o’-cobs^®^) was supplied by Envigo Laboratories, Inc. (Madison, WI). The Litron *In Vivo* Micronucleus Kit (MicroFlow^BASIC^ (Rodent Fixed Blood), Rochester, NY) was used to process the blood samples. Analyses of the food and water were conducted and recorded on a regular basis and the potential for known contaminants in the food or water was minimal and therefore unlikely to impact the outcomes of the studies.

### 2.3 Animals and organisms

The 14-day palatability/toxicity study was performed at Product Safety Labs (PSL) (Dayton, NJ). CRL Sprague-Dawley CD^®^ IGS rats (male and female) were purchased from Charles River Laboratories, Inc. (Raleigh, NC). The rats were young adults (approximately 7 weeks of age) with males weighing 192–248 g and females (nulliparous and non-pregnant) weighing 159–194 g at the start of the experiment. Only healthy rats without clinical signs of disease or injury and having adequate body weight gain and body weight (±20% of the mean) were included in the study. Animals were randomly assigned to cages/groups, stratified by body weight, on the day the study began. Individual animals were housed in suspended stainless steel cages with sizes in compliance with National Research Council [4] guidelines and litter paper was located below the cages, which was replaced a minimum of three times *per* week. A cage card was added to each cage, which indicated at minimum the study number, dose level, group assignment, individual animal identification (*i.e.,* sequential number plus a unique Monel^®^ stainless steel ear tag), and sex of the animal. The room in which the animals were housed maintained a temperature of 19–22 °C with 48–59% relative humidity and a 12-h light/dark cycle. The animals were acclimated to the housing conditions for five days prior to study initiation. The control and treatment diets (Research Diets, Inc., New Brunswick, NJ) and filtered tap water were supplied to the animals *ad libitum* throughout the study.

The 90-day dietary toxicity study was performed at PSL. CRL Sprague-Dawley CD^®^ IGS rats (male and female) were purchased from Charles River Laboratories, Inc. (Raleigh, NC). The rats were young adults (7–8 weeks of age) with males weighing 202–243 g and females (nulliparous and non-pregnant) weighing 156–183 g at the start of the experiment. Prior to study initiation, three sentinel rats were tested for common rat pathogens and the results were negative, therefore the study animals were considered to be healthy and reasonably free of common rat pathogens. Only rats free of clinical signs of disease or injury and having a body weight ±20% of the mean were selected for the study. Animals were randomly distributed to cages/groups, stratified by body weight, on the day the study began. Animals were housed individually in suspended stainless steel cages with sizes in compliance with National Research Council [4] guidelines and litter paper was located below the cages and replaced a minimum of three times *per* week. A cage card was added to each cage, which indicated at minimum the study number, dose level, group assignment, individual animal identification (*i.e.,* sequential number plus a unique Monel^®^ stainless steel ear tag), and sex of the animal. The room in which the animals were housed maintained a temperature of 19–23 °C with 38–72% relative humidity and a 12-h light/dark cycle. The humidity reached 72% on two days of the study, and a portable dehumidifier was used to reduce the humidity. The animals were acclimated to the housing conditions for six days prior to study initiation. The control and treatment diets (Research Diets, Inc.) and filtered tap water were supplied to the animals *ad libitum* throughout the study.

For the *in vitro* bacterial reverse mutation assay performed at PSL, *Salmonella typhimurium* (TA1535, TA1537, TA98, and TA100) and *Escherichia coli* WP2 uvrA were purchased from Molecular Toxicology, Inc.

For the *in vivo* mammalian erythrocyte micronucleus test performed at PSL, Swiss albino (ICR) mice were purchased from Envigo Laboratories, Inc. (Frederick, MD). The mice were young adults (7–8 weeks of age) with males weighing 31–39 g and females (nulliparous and non-pregnant) weighing 23–29 g at the start of the experiment. Animals were indiscriminately allocated to cages/groups as such there were no statistically significant differences in group body weights within a sex, and the animals were selected for the study on the basis that they were without clinical signs of disease or injury with a body weight ±20% of the mean body weight within a sex. Animals were housed in groups in solid bottom cages with sizes in compliance with National Research Council [4] guidelines and corncob-based bedding (Envigo Laboratories, Inc., Madison, WI). A cage card including the study number, dose level, group assignment, individual animal identifications (*i.e.,* a sequential number plus a unique marker (*e.g.,* ear tag or color marking)), and sex of the animals. The room in which the animals were housed maintained a temperature of 19–23 °C with 45–56% relative humidity and a 12-h light/dark cycle. There were 13 room air changes *per* hour throughout the duration of the study and airflow measurements were evaluated and recorded regularly. The animals were acclimated to the housing conditions for a period of five days prior to study initiation. Food (Harlan Teklad Global 16% Protein Rodent Diet^®^ #2016; Envigo Teklad Laboratories) and filtered tap water were supplied to the animals *ad libitum*.

### 2.4 Guidelines

Each study was conducted in compliance with the applicable OECD guideline [[5], [6], [7], [8]].

## 3 Experimental design

### 3.1 14-day palatability/toxicity study

Male and female CRL Sprague-Dawley CD^®^ IGS rats were administered KALGAE™ for a minimum of 14 days to evaluate the palatability and potential toxicity of KALGAE™. The animals were randomly assigned to receive either the basal (control) diet (*N* = 5 animals/sex) or one of three treatment diets (*N* = 5 animals/sex/dose) containing 50,000 ppm, 100,000 ppm, or 200,000 ppm KALGAE™. KALGAE™ was administered in the diet, because it is the proposed route of human exposure and recommended by OECD Guideline 407. The control and treatment diets (Research Diets, Inc.) and filtered tap water were supplied to the animals *ad libitum* throughout the study.

At least twice daily, the animals were observed for mortality, and cage-side observations were performed daily. A detailed clinical observation was conducted and recorded on Day 0 and weekly throughout the study. Individual body weights were measured and recorded at least twice during acclimation, on Day 0 (prior to study start), and throughout the study (Days 3, 7, 10, and 14). Body weight gain was determined for the selected intervals and the study overall. Individual food consumption and food efficiency were measured and recorded in parallel with body weight measurements, and the dietary intake of KALGAE™ was calculated. At the conclusion of the study, all surviving animals were euthanized by carbon dioxide asphyxiation and subjected to gross necropsy. All gross lesions were recorded. Animals were not fasted prior to necropsy.

### 3.2 90-day dietary toxicity study

Male and female CRL Sprague-Dawley CD^®^ IGS rats were administered KALGAE™ for a minimum of 90 days to evaluate the potential toxicity of KALGAE™. Based on the results of the 14-day palatability/toxicity study and ingredient availability, animals were randomly assigned to receive either the control diet (Group 1; *N *= 10 animals/sex) or a treatment diet (*N *= 10 animals/sex/dose) containing 37,500 ppm (Group 2), 75,000 ppm (Group 3), or 150,000 ppm (Group 4) KALGAE™. KALGAE™ was administered in the diet because it is the proposed route of human exposure and recommended by OECD Guideline 408. The control and treatment diets (Research Diets, Inc.) as well as filtered tap water were provided to the animals *ad libitum*.

Once during the acclimation period and prior to termination of the study (Day 79), all animals underwent an ophthalmologic evaluation by focal illumination, indirect ophthalmoscopy, and slit-lamp microscopy. Mydriatic eye drops were administered prior to the ophthalmoscopy and the eyes were examined in subdued light. At least twice daily animals were observed for mortality, and cage-side observations were performed daily. A detailed clinical observation was conducted on Day 0 and weekly throughout the study. Individual body weights were measured and recorded at least twice during acclimation, on Day 0, and weekly throughout the study (Days 7, 14, 21, 28, 35, 42, 49, 56, 63, 70, 77, 84, and 91). All surviving animals were weighed prior to sacrifice to allow for organ-to-body weight ratio calculations. Body weight gain was determined for the selected intervals and the study overall. Daily food consumption and food efficiency were measured and recorded for individual animals, and the dietary intake of KALGAE™ was calculated. Blood samples from all surviving animals were collected for clinical chemistry (Day 78), hematology (Day 78), and coagulation (Day 93). The animals were fasted overnight prior to sampling. The hematology and clinical chemistry blood samples were collected *via* sublingual bleeding under isoflurane anesthesia, and the blood samples for coagulation were collected *via* the inferior vena cava under isoflurane anesthesia at sacrifice. On Day 77, animals were placed in metabolism cages and were fasted for ≥15 h prior to urine collection on Day 78. From each surviving animal, urine was collected for urinalysis. All surviving animals were euthanized by exsanguination from the abdominal aorta under isoflurane anesthesia and subjected to gross necropsy. The following tissues were weighed (wet basis) and organ-to-body weights and organ-to-brain weights were recorded: adrenals (combined), kidneys (combined), testes (combined), brain, liver, thymus, epididymides (combined), ovaries with oviducts (combined), uterus, heart, and spleen. All organs and tissues from the surviving animals were preserved in 10% neutral buffered formalin for possible future histopathological examination, excluding the eyes, epididymides, optic nerve, and testes, which were preserved in modified Davidson’s fixative then stored in ethanol. The preserved organs and tissues from the control (Group 1) and high-dose (Group 4) groups were included in the histological examination. Additionally, histological examination was conducted for all tissues and organs with macroscopic observations for all animals. Tissues for all decedent were also evaluated. A board-certified veterinary pathologist prepared the slides and conducted the histological assessment at Histo-Scientific Research Laboratories (HSRL) (Frederick, MD).

### 3.3 *In vitro* bacterial reverse mutation assay

An *in vitro* bacterial reverse mutation assay was conducted to evaluate the potential for KALGAE™ to induce gene mutations in bacteria. Amino-acid requiring strains of *S. typhimurium* and *E. coli* were used to detect point mutations involving substitution, addition, or deletion of one or more DNA base pairs *via* the ability to functionally reverse mutations. Reverse mutations result in revertant colonies of bacteria that have a restored capability to synthesize the essential amino acids histidine and tryptophan. The S9 liver fraction, sourced from male Sprague-Dawley rats, was used in the assay to convert any potential promutagens into active metabolites capable of damaging DNA.

Using the standard plate incorporation method, the potential mutagenicity of KALGAE™ with and without S9 metabolic activation was evaluated in *S. typhimurium* TA1535, TA1537, TA98, and TA100 and *E. coli* WP2 uvrA (Molecular Toxicology, Inc.) (Experiment I). The pre-incubation modification of the plate incorporation test method was utilized for the confirmatory test (Experiment II). *Per* OECD Guideline 471, the maximum concentration used in the experiments was 5000 μg/plate. The standard plate incorporation method was conducted at concentrations of 0, 1.58, 5.0, 15.8, 50, 158, 500, 1580, or 5000 μg/plate with and without S9 metabolic activation using sterile water for serial dilutions (control vehicle) (Experiment I). The pre-incubation test (Experiment II) was performed at concentrations of 0, 1.58, 5.0, 15.8, 50, 158, 500, 1580, or 5000 μg/plate. The bacterial suspension cultures in nutrient broth were in the late exponential phase of growth with approximately 1 × 10^9^ bacteria/mL (100 μL/plate). The positive control substances in the absence of S9 metabolic activation included sodium azide (NaN_3_) (*S. typhimurium* TA1535 and TA100), ICR 191 acridine (*S. typhimurium* TA1537), daunomycin (*S. typhimurium* TA98), methyl methanesulfonate (MMS) (*E. coli* WP2 *uvr*A), 2-aminoanthracene (2-AA) (*S. typhimurium* TA1535, TA1537, TA98, and TA100 and *E. coli* WP2 uvrA) (Molecular Toxicology, Inc.). All treatments were performed and evaluated in triplicate.

For results to be considered positive and therefore indicative of mutagenic potential, the results had to show: (1) a substantial increase in revertant colony counts (*i.e.,* a response mutation factor ≥2 for *S. typhimurium* TA98 and TA100 and *E. coli* WP2 uvrA and a response mutation factor ≥3 for *S. typhimurium* TA1535 and TA1537) with mean value(s) outside of the laboratory historical control range; and (2) the increase in mutations had to be dose related and/or reproducible (*i.e.,* an increase must be obtained at more than one experimental point, in at least one strain, at more than one dose level, on more than one occasion, or with different methodologies). If the second criteria was not met, the results were reported as equivocal. If the test substance produced neither a concentration related increase in the number of revertant colonies nor a reproducible substantial increase in revertant colonies, then the test substance was considered to be non-mutagenic under the conditions of the study.

### 3.4 *In vivo* mammalian erythrocyte micronucleus test

A mammalian erythrocyte micronucleus test was conducted using Swiss albino (ICR) mice (Envigo Laboratories, Inc., Frederick, MD) to evaluate the *in vivo* genotoxic potential of KALGAE™. If a chromosome or spindle apparatus in dividing erythroblasts in the bone marrow is damaged, acentric fragments or lagging chromosomes condense in the cytoplasm to form micronuclei. As erythrocytes mature, the main nucleus is expelled but micronuclei tend to remain behind. Newly formed or immature micronucleated erythrocytes migrate to peripheral blood where they are easy to identify and readily quantifiable. The micronucleated erythrocytes are not efficiently removed from the circulating blood in mice, making mouse peripheral blood suitable for analysis.

A preliminary test with a negative (vehicle) control and a test substance group (*N = *3 animals/sex/group) was conducted to establish a maximum tolerated dose (MTD). A main test was then performed with three test groups (*N = *5 animals/sex/group), a negative control group (*N = *5 animals/sex), and a positive control group (*N = *5 animals/sex). KALGAE™ and the negative (vehicle) and positive control substances were administered *via* oral gavage, because it is a common route of human exposure and required by OECD Guideline 474. Bias was controlled for through the use of general procedures associated with balanced design and conduct of the study.

From the preliminary test, a MTD of 2000 mg KALGAE™/kg bw/day (delivered at a constant volume of 20 mL/kg) was established, which was the highest dose tested as recommended *per* OECD Guidelines, as it did not cause mortality or bone marrow toxicity. These data were used to select the doses of 500, 1,000, or 2000 mg KALGAE™/kg bw/day (25, 50, or 100% of the MTD and Groups 2, 3, and 4, respectively) delivered at a constant volume of 20 mL/kg for the main test. A negative (vehicle) control (distilled water delivered at a constant volume of 20 mL/kg; Group 1) and positive control (40 mg/kg bw/day cyclophosphamide monohydrate delivered at a constant volume of 5 mL/kg; Group 5) were also included in the main test. The test substance and negative (vehicle) control were delivered at a constant volume of 20 mL/kg due to the limited solubility of the test substance and were delivered in divided doses, two gavage treatments/day. All treatments were prepared on the day of dosing and the dosing formulations were maintained at room temperature. The positive control substance was administered *via* gavage on Day 2 of the study only, whereas the test substance and negative (vehicle) control were administered *via* gavage on Day 1 and Day 2. A stainless steel ball-tipped gavage needle attached to a syringe was used for the gavage treatment. Dosing occurred at approximately the same time each day ±2 h. At least twice *per* day animals were observed for mortality, and cage-side observations were performed and recorded daily during the study. The observations were conducted at intervals appropriate to assess the onset and termination of adverse effects, if any effects were observed. Approximately 30 min post-administration, the first observation was completed. Additional observations were also made and recorded during the first several hours post-administration on Day 1 for animals in the KALGAE™ treatment groups and negative (vehicle) control group. After the treatment period, animals were anesthetized with carbon dioxide (CO_2_), and whole blood was collected *via* cardiac puncture and processed according to the instructions in the Litron (*In vivo*) Micronucleus Kit (Rochester, NY). Following anesthesia, the animals were euthanized by exsanguination and discarded. In order to deem the results positive, there must have been a statistically significant increase in micronucleated immature erythrocyte (MIE) values as compared to the negative (vehicle) control group. Flow cytometry procedures were conducted at Litron Laboratories (Rochester, NY), and bone marrow slides were read by a board-certified veterinary pathologist.

## 4 Statistical analysis

### 4.1 14-day palatability/toxicity study

Statistical analyses were performed by PSL on all data collected during the in-life phase of the study and on organ weight data, if applicable. Statistical significance was judged at the 5% level. Mean and standard deviations were calculated for all quantitative data, with male and female rats evaluated separately. The statistical analyses were conducted using Provantis^®^ version 9 by Instem LSS (Staffordshire, UK). If required by the size of groups, data within groups were evaluated for homogeneity of variance and normality by Bartlett’s test [9]. If Barlett’s test supported homogeneous variances, a one-way ANOVA was used to compare the treatment and control groups. When the one-way ANOVA was significant, Dunnett’s test was used to compare the control and treatment groups [10,11]. If variances were considered significantly different by Bartlett’s test, the non-parametric method (*e.g.*, Kruskal–Wallis non-parametric analysis of variance) was applied [12]. If the non-parametric analysis was significant, the control and treatment groups were compared using Dunn’s test [13].

### 4.2 90-day dietary toxicity study

Statistical analyses were performed by PSL on all data collected during the in-life phase of the study and on organ weight data. The analysis of the clinical pathology results was conducted by DuPont Haskell Global Centers for Health and Environmental Sciences (Newark, DE) and provided to PSL. Statistical significance was judged at the 5% level. Mean and standard deviations were calculated for all quantitative data (in-life phase, organ weight, and clinical pathology parameters data), with male and female rats evaluated separately. The statistical analyses were conducted using Provantis^®^ version 9 by Instem LSS. For in-life data identified as multiple measurements of continuous data over time (*e.g.,* body weight parameters, food consumption, and food efficiency), all test groups were compared using two-way analysis of variance (ANOVA), testing the effects of both time and treatment [14]. Significant interactions between treatment and time, as well as main effects, were further analyzed by a *post hoc* multiple comparisons (*e.g*., Dunnett’s test). For organ weight data (absolute and relative), Bartlett’s test was used to evaluate homogeneity of variances and normality within groups, when the groups were of sufficient size. When homogeneous variance and normal distribution were observed, a one-way ANOVA was used to compare the treatment and control groups. When the one-way ANOVA was significant, a multiple comparisons test was used to compare the control and treatment groups (*e.g.,* Dunnett’s test [10,11]. If variances were significantly different, the control and treatment groups were evaluated using a non-parametric method (*e.g.,* Kruskal–Wallis non-parametric analysis of variance). If the non-parametric analysis was significant, the control and treatment groups were compared using Dunn’s test. For the clinical pathology data, Levene’s test for homogeneity and Shapiro–Wilk test for normality were used. If the tests were not significant, a one-way ANOVA followed by Dunnett’s test was used. If the tests were significant, transforms of the data (in the following order: log, square root, and rank-order) were used to achieve normality and variance homogeneity.

### 4.3 *In vitro* bacterial review mutation assay

Means and standard deviations were calculated for all quantitative data collected for the *in vitro* bacterial reverse mutation assay.

### 4.4 *In vivo* mammalian erythrocyte micronucleus test

For the *in vivo* mammalian erythrocyte micronucleus test, the proportions of immature erythrocytes among total erythrocytes and micronucleated erythrocytes were evaluated using ANOVA followed by Bonferroni-corrected multiple comparison test using PRISM Biostatistics, GraphPad Software (San Diego, CA).

## 5 Results

### 5.1 Dietary preparation and sampling

#### 5.1.1 14-day palatability/toxicity study

The stability of KALGAE™ in the dietary matrix was analyzed after 10 days of storage and determined to be 104.4% of the nominal concentration of 50,000 ppm KALGAE™ and 103.8% of the nominal concentration of 200,000 ppm KALGAE™. KALGAE™ was considered to be homogenously distributed in the diet at all nominal concentrations. The relative standard deviation was 1.83 and 2.81% between the strata for nominal concentrations of 50,000 and 200,000 ppm KALGAE™, respectively. The average percent of target concentrations on Day 0 were 104.5 and 104.8% of the target nominal concentrations of 50,000 and 200,000 ppm KALGAE™, respectively.

#### 5.1.2 90-day dietary toxicity study

*Beta*-carotene was utilized as a marker compound for the amount of KALGAE™ added to the diet. KALGAE™ (neat test substance) contained 98.1% of the target concentration of *beta*-carotene on Day 0 and 88.3% on Day 84. The overall stability of the neat test substance was determined to be 90% and considered to be within an acceptable margin of variation. The stability of KALGAE™ in the dietary matrix was analyzed after 4, 7, and 10 days of storage and determined to be 95.1, 96.3, and 97.9% on Day 4; 93.4, 93.6, and 94.2% on Day 7; and 79.1, 79.3, and 79.1% on Day 10 at nominal concentrations of 37,500, 75,000, and 150,000 ppm KALGAE™, respectively. The stability of *beta*-carotene in KALGAE™ in the dietary matrix was 97.6, 96.9, and 97.5% over seven days at nominal concentrations of 37,500, 75,000, and 150,000 ppm KALGAE™, respectively.

KALGAE™ was considered to be homogenously distributed in the diet at all nominal concentrations. On Day 0, the relative standard deviation was 5.62, 2.00, and 2.65% for nominal concentrations of 37,500, 75,000, and 150,000 ppm KALGAE™, respectively. The average target concentration percentages in the samples were 95.8, 96.6, and 96.6% of the target nominal concentrations of 37,500, 75,000, and 150,000 ppm KALGAE™, respectively.

The concentration of KALGAE™ was verified by analyzing *beta*-carotene in KALGAE™ on Day 0, Day 42, and Day 84. The mean samples were 95.8, 96.6, and 96.6% on Day 0, 89.4, 93.3, and 93.0% on Day 42, and 82.0, 76.8, and 83.9% on Day 84 for nominal concentrations of 37,500, 75,000, and 150,000 ppm KALGAE™, respectively.

### 5.2 Study results

#### 5.2.1 14-day palatability/toxicity study

There were no mortalities or clinical observations attributable to KALGAE™ in male or female rats (data not shown). Mean weekly body weight, mean daily body weight gain, mean daily food consumption, and mean daily food efficiency were not impacted by dietary intakes of KALGAE™ in any treatment group in male or females rats (data not shown). There were no abnormal gross necropsy observations in any study animals. The mean overall daily intake of KALGAE™ was 4086.4, 8108.9, or 17,166.4 mg/kg bw/day for males and 3796.9, 8115.0, or 15,486.3 mg/kg bw/day for females when fed dietary concentrations of 50,000, 100,000, or 200,000 ppm KALGAE™, respectively.

#### 5.2.2 90-day dietary toxicity study

No mortality related to the administration of KALGAE™ was observed in male or female rats. One male rat in Group 3 was found dead on Day 56. The animal was observed to have dark feces prior to death. Necropsy showed gastrointestinal distention and dark red discoloration of the spleen and liver, but there were no microscopic findings. The cause of death could not be determined by histopathological evaluation, but due to the absence of toxicological effects in the other rats in the mid- (Group 3) and high-dose (Group 4) treatment groups, the death was not attributed to exposure to KALGAE™.

**Fig. 1:**
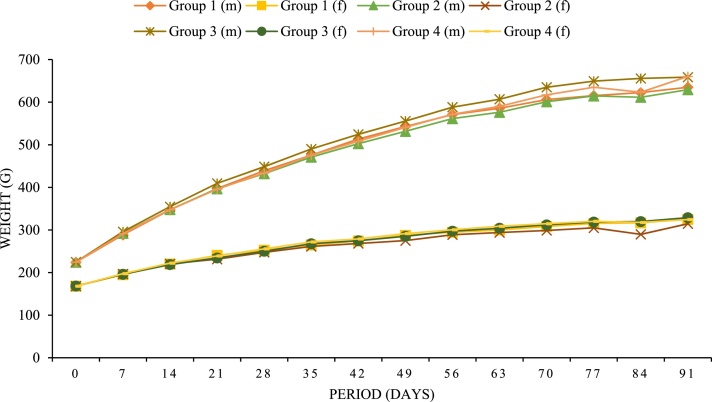
**Mean body weights of male (m) and female (f) rats consuming diets containing KALGAE™**. Markers on graph represent mean weight of respective group; basal (control) diet (Group 1; *N* = 10 animals/sex) or one of three treatment diets (*N* = 10 animals/sex/dose) containing 37,500 ppm (Group 2), 75,000 ppm (Group 3), or 150,000 ppm (Group 4).

There were no clinical signs, detailed clinical observations, or changes in body weight or body weight gain in male and female rats attributed to exposure to KALGAE™. For males, the mean weekly body weight and mean daily body weight gain for animals in the treatment groups were not statistically significant (*p > *0.05) from the control group (Fig. 1). In females, the mean weekly body weight for animals in the treatment groups were not statistically significant (*p > *0.05) from the control group (Fig. 1). The mean daily body weight gain of females in all treatment groups was comparable to the control group, except for an incidental, statistically significant (*p* < 0.05) decrease in Group 2 females on Days 42-49. The mean food consumption was not statistically significantly different in males and females in the treatment groups as compared to controls. The mean food efficiency for males and females in the treatments groups was not statistically significantly different than the control group, with the exception of females in Group 2, which had an incidental statistically significant (*p* < 0.05) decrease in mean food efficiency on Days 42–49 as compared to the control group. The body weight and food consumption data were used to calculate the daily intake of KALGAE™. In male rats, the mean nominal overall daily intake (Days 0–91) was calculated to be 1872.9, 3795.8, or 7895.2 mg KALGAE™/kg bw/day when fed dietary concentrations of 37,500, 75,000, or 150,000 ppm KALGAE™, respectively. In female rats, the mean nominal overall daily intake (Days 0–91) was calculated to be 2492.7, 4691.6, or 9708.09 mg KALGAE™/kg bw/day when fed dietary concentrations of 37,500, 75,000, or 150,000 ppm KALGAE™, respectively.

The ophthalmological examination (Day 79) identified one male rat in Group 4 with chorioretinal scarring and tapetal hyper-reflectivity in the left eye. The lesion was consistent with previous inflammation, likely due to vitreoretinal hemorrhage, which is common in young Sprague Dawley rats, therefore the effect was not attributed to exposure to KALGAE™.

There were no test-substance related effects observed in the hematology parameters assessed in male or female rats (Table 2 (male); Table 3 (female)). On Day 78, Group 4 male rats had statistically significantly increased (*p* < 0.05) mean corpuscular hemoglobin (MCH) and decreased (*p* < 0.05) red cell distribution width (RDW). Females in the Group 3 and Group 4 also had statistically significantly decreased RDW (*p* < 0.05). Because the changes were not associated with histopathological changes or changes in red blood cell parameters and were within historical control range values for the PSL facility for this study period, the changes were not considered toxicologically significant. In the Group 2 and Group 4 male rats, there was a statistically significant (*p* < 0.05) decrease in absolute neutrophils (ANEU). This result was not considered to be toxicologically significant because total blood cell counts were unchanged, there were no accompanying histopathological effects or dose-response effect, and the ANEU result was within laboratory historical control range values.

**Table 2:** Hematology and coagulation parameters in male rats.

Parameter	0 ppm Group 1 *N* = 9	37,500 ppm Group 2 *N* = 10	75,000 ppm Group 3 *N* = 9	150,000 ppm Group 4 *N* = 10
Hematology, 78 days relative to start date				
RBC (x10^6^/μL)	8.73 ± 0.37	8.65 ± 0.23	8.73 ± 0.34	8.43 ± 0.60
HGB (g/dL)	15.1 ± 0.4	15.5 ± 0.6	15.5 ± 0.6	15.3 ± 0.9
HCT (%)	45.2 ± 1.2	46.0 ± 1.3	46.2 ± 1.8	44.9 ± 3.2
MCV (fL)	51.8 ± 1.4	53.2 ± 1.0	52.9 ± 1.9	53.3 ± 1.3
MCH (pg)	17.3 ± 0.5	17.9 ± 0.6	17.8 ± 0.8	18.2 ± 0.7*
MCHC (g/dL)	33.5 ± 0.3	33.6 ± 0.5	33.5 ± 0.5	34.1 ± 0.8
RDW (%)	12.8 ± 0.5	12.7 ± 0.6	12.6 ± 0.4	11.9 ± 0.5*
PLT (x10^3^/μL)	861 ± 117	887 ± 123	925 ± 110	894 ± 107
WBC (x10^3^/μL)	12.48 ± 2.61	13.14 ± 2.46	13.82 ± 3.25	11.94 ± 3.04
ANEU (x10^3^/μL)	3.37 ± 1.10	2.17 ± 0.82*	2.53 ± 1.15	1.58 ± 0.35*
ALYM (x10^3^/μL)	8.41 ± 2.43	10.22 ± 2.53	10.46 ± 3.18	9.75 ± 2.79
AMON (x10^3^/μL)	0.38 ± 0.15	0.32 ± 0.13	0.38 ± 0.11	0.28 ± 0.11
AEOS (x10^3^/μL)	0.17 ± 0.07	0.22 ± 0.08	0.20 ± 0.06	0.18 ± 0.04
ABAS (x10^3^/μL)	0.04 ± 0.02	0.07 ± 0.04	0.08 ± 0.10	0.06 ± 0.04
ALUC (x10^3^/μL)	0.12 ± 0.07	0.14 ± 0.05	0.18 ± 0.06	0.09 ± 0.06
ARET (x10^3^/μL)	197.5 ± 22.9	191.8 ± 29.1	210.6 ± 30.3	186.0 ± 26.2

Coagulation, 92 days relative to start date				
PT (sec)	10.6 ± 0.3	11.0 ± 0.4*	10.9 ± 0.2	11.3 ± 0.3*
APTT (sec)	24.7 ± 4.9	25.1 ± 4.4	26.1 ± 7.5	25.6 ± 4.3

ABAS, absolute basophils; AEOS, absolute eosinophils; ALUC, absolute large unstained cells; ALYM, absolute lymphocytes; AMON, absolute monocytes; ANEU, absolute neutrophils; APTT, activated partial thromboplastin time; ARET, absolute reticulocytes; HCT, hematocrit; HGB, hemoglobin; MCH, mean corpuscular hemoglobin; MCHC, mean corpuscular hemoglobin concentration; MCV, mean corpuscular volume; PLT, platelet count; ppm, parts *per* million; PT, prothrombin time; RBC, erythrocytes; RDW, red cell distribution width; WBC, total white blood cells; Mean ± SD shown; *p < 0.05 *vs*. Group 1 (control).

**Table 3:** Hematology and coagulation parameters in female rats.

Parameter	0 ppm Group 1 *N* = 10	37,500 ppm Group 2 *N* = 10	75,000 ppm Group 3 *N* = 10	150,000 ppm Group 4 *N* = 10
Hematology, 78 days relative to start date				
RBCx10^6^/μL)	8.07 ± 0.21	8.24 ± 0.27	8.05 ± 0.27	8.02 ± 0.29
HGB (g/dL)	14.6 ± 0.6	15.0 ± 0.4	14.8 ± 0.5	14.7 ± 0.6
HCT (%)	43.0 ± 1.4	44.2 ± 1.4	43.4 ± 1.5	42.9 ± 1.5
MCV (fL)	53.2 ± 1.7	53.7 ± 1.7	53.9 ± 1.5	53.5 ± 1.5
MCH (pg)	18.1 ± 0.7	18.2 ± 0.5	18.3 ± 0.6	18.4 ± 0.6
MCHC (g/dL)	34.0 ± 0.5	33.9 ± 0.4	34.0 ± 0.4	34.3 ± 0.5
RDW (%)	11.9 ± 0.4	11.6 ± 0.4	11.3 ± 0.4*	11.4 ± 0.5*
PLT (x10^3^/μL)	931 ± 127	875 ± 65	928 ± 55	944 ± 78
WBC (x10^3^/μL)	7.45 ± 1.72	7.15 ± 1.77	7.32 ± 1.93	6.01 ± 1.39
ANEU (x10^3^/μL)	1.54 ± 0.56	1.18 ± 0.61	1.11 ± 0.46	0.99 ± 0.35
ALYM (x10^3^/μL)	5.54 ± 1.42	5.61 ± 1.56	5.84 ± 1.66	4.73 ± 1.23
AMON (x10^3^/μL)	0.15 ± 0.08	0.17 ± 0.07	0.17 ± 0.10	0.12 ± 0.03
AEOS (x10^3^/μL)	0.13 ± 0.07	0.11 ± 0.04	0.10 ± 0.02	0.10 ± 0.03
ABAS (x10^3^/μL)	0.03 ± 0.01	0.01 ± 0.01	0.03 ± 0.02	0.02 ± 0.02
ALUC (x10^3^/μL)	0.06 ± 0.02	0.07 ± 0.03	0.07 ± 0.06	0.05 ± 0.03
ARET (x10^3^/μL)	183.5 ± 39.4	168.4 ± 29.3	153.9 ± 25.3	161.4 ± 31.0

Coagulation, 93 days relative to start date				
PT (s)	10.2 ± 0.2	10.2 ± 0.1	10.3 ± 0.2	10.2 ± 0.2
APTT (s)	24.4 ± 3.5	26.5 ± 4.6	27.1 ± 5.4	23.9 ± 4.2

ABAS, absolute basophils; AEOS, absolute eosinophils; ALUC, absolute large unstained cells; ALYM, absolute lymphocytes; AMON, absolute monocytes; ANEU, absolute neutrophils; APTT, activated partial thromboplastin time; ARET, absolute reticulocytes; HCT, hematocrit; HGB, hemoglobin; MCH, mean corpuscular hemoglobin; MCHC, mean corpuscular hemoglobin concentration; MCV, mean corpuscular volume; PLT, platelet count; ppm, parts *per* million; PT, prothrombin time; RBC, erythrocytes; RDW, red cell distribution width; WBC, total white blood cells; Mean ± SD shown; *p < 0.05 *vs*. Group 1 (control).

The coagulation parameters assessed did not show any effects related to exposure to KALGAE™ in male or female rats (Table 2 (male); Table 3 (female)). On Day 92, males in Group 2 and Group 4 had statistically significantly (*p* < 0.05) increased prothrombin time (PT), but the change was of small magnitude, within laboratory historical control range values, and not associated with histopathological or clinical signs. Therefore, the PT change was not considered to be toxicologically relevant or treatment-related. A coagulation sample was not collected from Animal No. 7003 due to the liver being damaged during blood collection.

The clinical chemistry parameters did not indicate any effects related to exposure to KALGAE™ in male or female rats (Table 4 (male); Table 5 (female)). On Day 78, statistically significant (*p* < 0.05) decreases in cholesterol, total serum protein, and globulin were noted in Group 4 male rats. The changes were within the laboratory historical control range values and not associated with histopathological changes, and therefore, were determined to not be of toxicological significance. Statistically significant (*p* < 0.05) decreases in alkaline phosphatase (ALKP) in Group 2 and Group 4 females were recorded but considered to not be of clinical relevance or toxicological significance. Statistically significant (*p* < 0.05) decreases in sorbitol dehydrogenase (SDH) in Group 2 males, serum alanine aminotransferase (ALT) in Group 2 females, and calcium in Group 3 females were non-dose dependent, within the laboratory’s historical control range values, and interpreted to be within expected biological variation, and therefore not considered to be of toxicological relevance.

**Table 4:** Clinical chemistry and urinalysis parameters in male rats (78 days relative to start date).

Clinical chemistry	0 ppm Group 1 *N* = 9-10	37,500 ppm Group 2 *N* = 10	75,000 ppm Group 3 *N* = 9	150,000 ppm Group 4 *N* = 9-10
AST (U/L)	186 ± 218	91 ± 33	102 ± 43	78 ± 13
ALT (U/L)	93 ± 137	39 ± 39	57 ± 63	32 ± 14
SDH (U/L)	27.6 ± 44.8	6.5 ± 5.7*	12.4 ± 9.7	7.4 ± 3.5
ALKP (U/L)	110 ± 36	103 ± 19	103 ± 13	116 ± 25
BILI (mg/dL)	0.19 ± 0.05	0.16 ± 0.02	0.18 ± 0.02	0.17 ± 0.02
BUN (mg/dL)	10 ± 2	10 ± 1	10 ± 1	10 ± 1
Creatinine (mg/dL)	0.23 ± 0.02	0.23 ± 0.02	0.23 ± 0.03	0.23 ± 0.03
Total cholesterol (mg/dL)	93 ± 22	80 ± 23	85 ± 27	65 ± 11*
Triglycerides (mg/dL)	76 ± 47	68 ± 22	82 ± 33	70 ± 14
Glucose, fasting (mg/dL)	126 ± 22	112 ± 14	118 ± 15	118 ± 16
Total protein (g/dL)	6.3 ± 0.2	6.1 ± 0.2	6.3 ± 0.3	6.0 ± 0.1*
Albumin (g/dL)	3.1 ± 0.1	3.1 ± 0.1	3.1 ± 0.2	3.0 ± 0.1
Globulin (g/dL)	3.2 ± 0.02	3.0 ± 0.1	3.1 ± 0.2	3.0 ± 0.1*
Calcium (mg/dL)	10.2 ± 0.3	10.1 ± 0.2	10.2 ± 0.4	10.1 ± 0.2
Inorganic phosphate (mg/dL)	6.7 ± 0.6	7.0 ± 0.6	7.0 ± 0.4	7.1 ± 0.5
Sodium (mmol/L)	140.5 ± 0.5	140.8 ± 1.1	140.8 ± 1.1	140.6 ± 1.0
Potassium (mmol/L)	5.04 ± 0.25	5.16 ± 0.20	5.01 ± 0.36	4.97 ± 0.17
Chloride (mmol/L)	101.4 ± 0.8	102.0 ± 0.7	101.3 ± 1.7	102.1 ± 1.1

Urinalysis	*N* = 10	*N* = 10	*N* = 9	*N* = 10
Urine volume (mL)	4.3 ± 2.7	4.8 ± 3.4	5.1 ± 4.1	7.3 ± 4.6
pH	6.2 ± 0.3	6.2 ± 0.3	6.2 ± 0.4	6.3 ± 0.4
Specific gravity	1.062 ± 0.026	1.052 ± 0.019	1.056 ± 0.019	1.047 ± 0.025
URO (EU/dL)	0.3 ± 0.3	0.2 ± 0.0	0.4 ± 0.4	0.4 ± 0.3
UMTP (mg/dL)	224 ± 108	199 ± 94	221 ± 153	167 ± 101

ALKP, alkaline phosphatase; ALT, alanine aminotransferase; AST, aspartate aminotransferase; BILI, total bilirubin; BUN, blood urea nitrogen; ppm, parts *per* million; SDH, sorbitol dehydrogenase; UMTP, protein; URO, urobilinogen; Mean ± SD shown; *p < 0.05 *vs*. Group 1 (control).

**Table 5:** Clinical chemistry and urinalysis parameters in female rats (78 days relative to start date).

Clinical chemistry	0 ppm Group 1 *N* = 10	37,500 ppm Group 2 *N* = 9-10	75,000 ppm Group 3 *N* = 10	150,000 ppm Group 4 *N* = 10
AST (U/L)	75 ± 16	75 ± 8	82 ± 13	73 ± 15
ALT (U/L)	23 ± 3	18 ± 3*	22 ± 6	19 ± 4
SDH (U/L)	5.7 ± 2.7	7.3 ± 4.7	5.3 ± 2.6	4.7 ± 1.5
ALKP (U/L)	75 ± 40	49 ± 10*	58 ± 16	48 ± 14*
BILI (mg/dL)	0.17 ± 0.03	0.18 ± 0.02	0.18 ± 0.03	0.19 ± 0.03
BUN (mg/dL)	12 ± 2	12 ± 1	12 ± 2	11 ± 2
Creatinine (mg/dL)	0.31 ± 0.02	0.32 ± 0.04	0.33 ± 0.03	0.31 ± 0.03
Total cholesterol (mg/dL)	80 ± 19	71 ± 19	71 ± 13	71 ± 17
Triglycerides (mg/dL)	43 ± 9	41 ± 11	40 ± 9	39 ± 17
Glucose, fasting (mg/dL)	121 ± 17	116 ± 7	123 ± 11	115 ± 11
Total protein (g/dL)	7.4 ± 0.4	7.1 ± 0.3	7.1 ± 0.3	7.3 ± 0.4
Albumin (g/dL)	4.1 ± 0.3	3.9 ± 0.2	3.9 ± 0.2	4.0 ± 0.3
Globulin (g/dL)	3.3 ± 0.2	3.2 ± 0.2	3.2 ± 0.1	3.3 ± 0.2
Calcium (mg/dL)	10.4 ± 0.4	10.2 ± 0.4	10.0 ± 0.2*	10.2 ± 0.3
Inorganic phosphate (mg/dL)	4.9 ± 0.9	4.7 ± 0.8	4.6 ± 0.6	4.5 ± 0.7
Sodium (mmol/L)	139.5 ± 1.0	139.8 ± 0.9	139.5	139.6 ± 1.3
Potassium (mmol/L)	4.37 ± 0.30	4.41 ± 0.35	4.63 ± 0.42	4.44 ± 0.26
Chloride (mmol/L)	101.8 ± 1.9	102.0 ± 1.8	102.7 ± 1.1	101.9 ± 1.7

Urinalysis	*N* = 10	*N* = 10	*N* = 9-10	*N* = 10
Urine volume (mL)	2.4 ± 1.1	1.4 ± 0.7	2.4 ± 2.2	3.7 ± 2.0
pH	6.2 ± 0.2	6.0 ± 0.2	6.2 ± 0.2	6.5 ± 0.6
Specific gravity	1.060 ± 0.019	1.076 ± 0.023	1.064 ± 0.026	1.042 ± 0.017
URO (EU/dL)	0.2 ± 0.0	0.2 ± 0.0	0.2 ± 0.0	0.2 ± 0.0
UMTP (mg/dL)	80 ± 27	126 ± 87	100 ± 58	61 ± 27

ALKP, alkaline phosphatase; ALT, alanine aminotransferase; AST, aspartate aminotransferase; BILI, total bilirubin; BUN, blood urea nitrogen; ppm, parts *per* million; SDH, sorbitol dehydrogenase; UMTP, protein; URO, urobilinogen; Mean ± SD shown; *p < 0.05 *vs*. Group 1 (control).

There were no changes related to the administration of KALGAE™ in the urinalysis parameter in male or female rats on Day 78 (Table 4 (male); Table 5 (female)). During the necropsy of male and female rats, no treatment-related lesions were identified. A fluid filled uterus was observed in three females in the control group (Group 1), one female in Group 2, one female in Group 3, and one female in Group 4. This finding was considered spontaneous and incidental and confirmed microscopically. The finding was also consistent with physiologic changes related to normal estrous cycling. In males, bilateral small testes and epididymides were found in one Group 2 male and one Group 3 male. The finding was considered spontaneous and incidental and confirmed microscopically as spontaneous testes atrophy with epididymides aspermia. One male in the control group (Group 1) had a unilateral tan epididymis nodule, which was confirmed microscopically as a spontaneous sperm granuloma. One male in the control group had an enlarged spleen and an apparent discontinuity of the dorsal palate. Microscopically, the male rat had a missing tooth, with bone fragments and inflammation leading to a palate defect, along with increased extramedullary hematopoiesis in the spleen, which often occurs in response to an inflammatory process. These findings were considered spontaneous and incidental.

There were no microscopic observations related to the dietary exposure of KALGAE™ in male or female rats. All changes present were consistent in morphology, severity, and incidence with spontaneous changes in young adult Sprague-Dawley rats.

Absolute organ weights and organ-to-body/brain weight ratios did not support any toxicologically relevant effects related to the dietary exposure of KALGAE™ in male and female rats (Table 6 (male); Table 7 (female)). Statistically significant (*p* < 0.01–0.05) increases in absolute kidney weights for Group 3 and Group 4 males and kidney-to-brain weight ratios in Group 4 males were observed. In Group 4 female rats, absolute spleen weights, spleen-to-body weight ratios, and spleen-to-brain weight ratios were significantly (*p* < 0.01) decreased. An incidental statistically significant (*p* < 0.05) decrease in liver-to-body weight ratio was noted in Group 3 females. These changes were not considered to be of toxicological relevance in the absence of clinical and histopathological changes.

**Table 6:** Summary of mean terminal body and organ weights in male rats (92 days relative to start date).

Parameter	0 ppm Group 1 *N* = 9-10	37,500 ppm Group 2 *N* = 10	75,000 ppm Group 3 *N* = 9	150,000 ppm Group 4 *N* = 10
Mean terminal body and organ weights (grams)				
Body Weight	611.8 ± 68.7	607.9 ± 73.9	648.2 ± 69.4	632.1 ± 47.3
Adrenal Glands	0.0574 ± 0.0122 I^1^	0.0645 ± 0.0106	0.0648 ± 0.0105	0.0627 ± 0.0062
Brain	2.286 ± 0.118 I^1^	2.277 ± 0.111	2.320 ± 0.076	2.281 ± 0.068
Epididymides	1.6433 ± 0.1381 R^2^	1.5485 ± 0.3064	1.5294 ± 0.2627	1.6258 ± 0.1818
Heart	1.640 ± 0.144 R^2^	1.610 ± 0.101	1.716 ± 0.188	1.711 ± 0.158
Kidneys	3.582 ± 0.258^#^	3.755 ± 0.435	4.027 ± 0.356*	4.128 ± 0.384**
Liver	15.166 ± 2.535 I^1^	14.626 ± 2.378	15.240 ± 1.917	15.486 ± 1.378
Spleen	0.896 ± 0.095 R^2^	0.784 ± 0.077	0.843 ± 0.090	0.790 ± 0.189
Testes	3.582 ± 0.250 R^2^	3.513 ± 0.875	3.371 ± 0.868	3.728 ± 0.258
Thymus	0.3011 ± 0.0672 I^1^	0.3413 ± 0.1014	0.3512 ± 0.1082	0.2953 ± 0.0777

Mean organ-to-body weight (ratio)				
Adrenal/TBW	0.0945 ± 0.0222 I^1^	0.1082 ± 0.0268	0.1009 ± 0.0190	0.0997 ± 0.0124
Brain/TBW	3.769 ± 0.363 I^1^	3.802 ± 0.536	3.617 ± 0.421	3.631 ± 0.346
Epididymides/TBW	2.7081 ± 0.3054 R^2^	2.5905 ± 0.6308	2.3804 ± 0.4623	2.5832 ± 0.3344
Heart/TBW	2.703 ± 0.327 I^1^	2.670 ± 0.219	2.655 ± 0.220	2.716 ± 0.284
Kidneys/TBW	5.891 ± 0.458 I^1^	6.200 ± 0.491	6.252 ± 0.629	6.565 ± 0.821
Liver/TBW	24.712 ± 2.093 I^1^	24.019 ± 2.229	23.533 ± 1.773	24.538 ± 1.872
Spleen/TBW	1.447 ± 0.135 I^1^	1.301 ± 0.156	1.309 ± 0.154	1.250 ± 0.279
Testes/TBW	5.911 ± 0.693 R^2^	5.890 ± 1.718	5.245 ± 1.436	5.926 ± 0.593
Thymus/TBW	0.4965 ± 0.1179 I^1^	0.5658 ± 0.1626	0.5437 ± 0.1592	0.4711 ± 0.1364

Mean organ-to-brain weight (ratio)				
Adrenal/BrW	0.0251 ± 0.0055 I^1^	0.0284 ± 0.0053	0.0280 ± 0.0051	0.0275 ± 0.0030
Epididymides/BrW	0.7208 ± 0.0739 I^1^	0.6826 ± 0.1454	0.6577 ± 0.1061	0.7135 ± 0.0856
Heart/BrW	0.718 ± 0.054 L^3^	0.709 ± 0.062	0.740 ± 0.084	0.751 ± 0.072
Kidneys/BrW	1.567 ± 0.086 ^	1.653 ± 0.212	1.736 ± 0.150	1.811 ± 0.172**
Liver/BrW	6.646 ± 1.154 I^1^	6.435 ± 1.090	6.572 ± 0.803	6.794 ± 0.636
Spleen/BrW	0.393 ± 0.048 R^2^	0.345 ± 0.036	0.364 ± 0.037	0.347 ± 0.086
Testes/BrW	1.570 ± 0.131 R^2^	1.546 ± 0.385	1.446 ± 0.360	1.636 ± 0.125
Thymus/BrW	0.1328 ± 0.0335 I^1^	0.1501 ± 0.0448	0.1511 ± 0.0459	0.1300 ± 0.0366

BrW, brain weight; TBW, terminal body weight.

I^1^ = Automatic transformation: Identity (No transformation).

R^2^ = Automatic transformation: Rank.

L^3^=Automatic transformation: Log.

^ = Automatic transformation: Identity (No transformation), (All groups) Test: Analysis of variance *p *< 0.05.

# = Automatic transformation: Identity (No transformation), (All groups) Test: Analysis of variance *p *< 0.01.

Mean ± SD shown; *p < 0.05 *vs*. Group 1 (control),** *p *< 0.01 *vs*. Group 1 (control).

**Table 7:** Summary of mean terminal body and organ weights in female rats (93 days relative to start date).

Parameter	0 ppm Group 1 *N* = 10	37,500 ppm Group 2 *N* = 10	75,000 ppm Group 3 *N* = 10	150,000 ppm Group 4 *N* = 10
Mean terminal body and organ weights (grams)				
Body weight	314.3 ± 26.9	300.8 ± 31.8	316.0 ± 33.5	309.4 ± 37.9
Adrenal glands	0.0729 ± 0.0101 R^1^	0.0817 ± 0.0128	0.1633 ± 0.2952	0.0757 ± 0.0127
Brain	2.074 ± 0.068 R^1^	2.074 ± 0.081	2.105 ± 0.105	2.049 ± 0.084
Heart	1.096 ± 0.060 R^1^	1.085 ± 0.155	1.047 ± 0.079	1.047 ± 0.135
Kidneys	2.267 ± 0.176 I^2^	2.107 ± 0.145	2.107 ± 0.173	2.179 ± 0.258
Liver	8.980 ± 0.920 I^2^	8.456 ± 0.919	8.105 ± 0.798	8.338 ± 1.395
Ovaries with oviducts	0.1430 ± 0.0270 L^3^	0.1319 ± 0.0298	0.1279 ± 0.0164	0.1320 ± 0.0192
Spleen	0.567 ± 0.079 ^#^	0.561 ± 0.085	0.515 ± 0.058	0.457 ± 0.080**
Thymus	0.2547 ± 0.0277 R^1^	0.2420 ± 0.0889	0.3030 ± 0.0848	0.2416 ± 0.0905
Uterus	0.765 ± 0.223 L^3^	0.682 ± 0.177	0.697 ± 0.303	0.704 ± 0.138

Mean organ-to-body weight (ratio)				
Adrenal/TBW	0.2333 ± 0.0360 R^1^	0.2753 ± 0.0566	0.4955 ± 0.8512	0.2488 ± 0.0529
Brain/TBW	6.635 ± 0.511 I^2^	6.969 ± 0.827	6.716 ± 0.624	6.710 ± 0.849
Heart/TBW	3.497 ± 0.161 ^	3.600 ± 0.238	3.332 ± 0.274	3.387 ± 0.190
Kidneys/TBW	7.232 ± 0.494 I^2^	7.045 ± 0.539	6.714 ± 0.675	7.066 ± 0.555
Liver/TBW	28.610 ± 2.279 ^	28.240 ± 2.860	25.723 ± 1.751*	26.882 ± 2.274
Ovaries with oviducts/TBW	0.4530 ± 0.0632 L^3^	0.4417 ± 0.1026	0.4091 ± 0.0699	0.4299 ± 0.0664
Spleen/TBW	1.800 ± 0.158 ^ǂ^	1.880 ± 0.324	1.633 ± 0.130	1.480 ± 0.199**
Thymus/TBW	0.8122 ± 0.0783 R^1^	0.7967 ± 0.2634	0.9593 ± 0.2435	0.7729 ± 0.2586
Uterus/TBW	2.439 ± 0.705 L^3^	2.273 ± 0.544	2.291 ± 1.264	2.339 ± 0.714

Mean organ-to-brain weight (ratio)				
Adrenal/BrW	0.0352 ± 0.0052 R^1^	0.0395 ± 0.0066	0.0754 ± 0.1330	0.0369 ± 0.0056
Heart/BrW	0.529 ± 0.025 R^1^	0.524 ± 0.082	0.498 ± 0.032	0.511 ± 0.067
Kidneys/BrW	1.092 ± 0.066 R^1^	1.018 ± 0.090	1.000 ± 0.050	1.065 ± 0.136
Liver/BrW	4.325 ± 0.362 R^1^	4.087 ± 0.504	3.845 ± 0.260	4.076 ± 0.707
Ovaries with oviducts/BrW	0.0689 ± 0.0122 I^2^	0.0635 ± 0.0130	0.0608 ± 0.0073	0.0644 ± 0.0088
Spleen/BrW	0.273 ± 0.033 ^#^	0.271 ± 0.044	0.245 ± 0.026	0.223 ± 0.037**
Thymus/BrW	0.1230 ± 0.0150 R^1^	0.1167 ± 0.0434	0.1441 ± 0.0396	0.1185 ± 0.0454
Uterus/BrW	0.369 ± 0.109 L^3^	0.330 ± 0.090	0.335 ± 0.156	0.345 ± 0.071

BrW, brain weight; TBW, terminal body weight.

R^1^ = Automatic transformation: Rank.

I^2^ = Automatic transformation: Identity (No transformation).

L^3^ = Automatic transformation: Log.

ǂ = Automatic transformation: Log, (All groups) Test: Analysis of variance *p *< 0.001.

# = Automatic transformation: Identity (No transformation), (All groups) Test: Analysis of variance *p *< 0.01.

^Automatic transformation: Identity (No transformation), (All groups) Test: Analysis of variance *p *< 0.05.

Mean ± SD shown; *p < 0.05 *vs*. Group 1 (control),** *p *< 0.01 *vs*. Group 1 (control).

#### 5.2.3 *In vitro* bacterial reverse mutation assay

There were no mutagenic effects observed in *S. typhimurium* TA1535, TA1537, TA98, or TA100 or *E. coli* WP2 uvrA with or without S9 metabolic activation using either the plate incorporation (Experiment I) or the pre-incubation method (Experiment II) (data not shown). Evidence of precipitation was noted in all bacterial strains ≥1580 μg/plate in Experiment I and II with and without S9 metabolic activation. At 5000 μg/plate, the precipitation impacted lawn evaluation for all strains; however, the precipitation did not obscure or impact revertant counts. A minimum of six dose levels without precipitation were evaluated, supporting the fact that mutagenicity was adequately assessed. There were no signs of contamination for any of the strains tested. The mean revertant colony counts observed for each strain treated with the vehicle fell near or inside of the anticipated range, when taking into consideration the laboratory historical control range and/or published values [15,16]. With and without S9 metabolic activation, increases in revertant colony counts were observed in each phase when treated with the positive control substances, supporting the sensitivity of the test and the activity of the S9. All tests were considered valid. There were no deviations to the study protocol.

There were no substantial increases in revertant colony counts in *S. typhimurium* TA98, TA100, TA1535, or TA1537 or *E. coli* WP2 uvrA outside of the laboratory historical control range with or without S9 metabolic activation. Also, there were no dose-related increases in mutations with or without S9 metabolic activation. The mean control responses were (with-without S9 metabolic activation): TA1535 (12–17), TA1537 (14–12), TA98 (29–30), TA100 (107–101), and *E. coli* WP2 uvrA (55–50). KALGAE™ did not produce a concentration-dependent increase in revertant colonies or a substantial reproducible increase in revertant colonies, therefore the test substance was considered to be non-mutagenic under the conditions of the study.

#### 5.2.4 *In vivo* mammalian erythrocyte micronucleus test

In the preliminary test, no mortality was reported, animals appeared healthy and active throughout the study, and the body weights for animals in the treatment group *versus* the control group were not significantly different on Day 1. The percentage of immature erythrocytes for the MTD limit dose of KALGAE™ were comparable to the control values. In the main test, no mortality was reported. The body weights of the animals at the initiation of the study were all within ±20% of the mean within a sex (data not shown). Superficial eschar was recorded in 3/5 males in Group 1 (negative (vehicle) control) and 1/5 males in Group 5 (positive control). All animals were active and healthy throughout the observation period. The positive control had a statistically significant decrease (*p* < 0.001–0.05) in the frequency of reticulocytes (%RET) (male), increase in the frequency of micronucleated normochromatic erythrocytes (%MN-NCE) (female), and increase in frequency of positive micronucleated reticulocytes (%MN-RET) (male and female) as compared to the negative (vehicle) control. In Groups 2, 3, and 4 which were administered KALGAE™, there were no test substance-related changes in %RET, %MN-MCE, or %MN-RET in the blood of male or female mice, as compared to the negative (vehicle) control (data not shown). All tests were considered valid. There was one deviation to the study protocol as the animals were weighed on Day 4, which was not required *per* the study protocol. The weights obtained on Day 4 were not used in the interpretation of the study results and were deemed to have no impact on the animals, consequently the weighing of the animals on Day 4 was deemed to have no impact on the study. KALGAE™ did not induce micronucleus formation in the immature erythrocytes of mice in this study, therefore KALGAE™ was not considered to be genotoxic under the conditions of this study.

## 6 Discussion

No treatment-related adverse effects were observed when KALGAE™ was administered to male and female CRL Sprague-Dawley CD^®^ IGS rats in the diet for 14 days at 4086.4, 8108.9, or 17,166.4 mg/kg bw/day in males and 3796.9, 8115.0, or 15,486.3 mg/kg bw/day in females. Dietary administration of KALGAE™ to male and female CRL Sprague-Dawley CD^®^ IGS rats for 90 days did not result in any treatment-related effects, even at the highest dose tested. One male rat in Group 3 was found dead on Day 56. The animal was observed to have dark feces prior to death. Necropsy showed gastrointestinal distention and dark red discoloration of the spleen and liver, but there were no microscopic findings. The cause of death could not be determined by histopathological evaluation, but due to the absence of toxicological effects in the other rats in Group 3 and Group 4, the death was not attributed to KALGAE™. No mortality related to the administration of KALGAE™ was observed in male or female rats.

There were no clinical signs, detailed clinical observations, or ophthalmological effects attributed to exposure to KALGAE™ in male or female rats. There were no changes in coagulation, clinical chemistry, urinalysis parameters, body weight, body weight gain, mean food consumption, or mean food efficiency in male and female rats attributable to the administration of KALGAE™. During the necropsy of male and female rats, no treatment-related lesions were identified. There were no microscopic observations attributed to the dietary exposure of KALGAE™ in male or female rats, as all changes present were consistent in morphology, severity, and incidence with spontaneous changes in young adult Sprague-Dawley rats.

There were no test-substance related effects observed in the hematology parameters assessed in male or female rats. The Group 4 male rats had statistically significantly increased MCH, and Group 4 males and Group 3 and 4 females had statistically significantly decreased RDW. According to the board-certified clinical pathologist, these changes in MCH and RDW were not considered to be adverse as they were not accompanied by changes in red blood cell (RBC) parameters (decreases in erythrocytes (RBC), hemoglobin (HGB), or hematocrit (HCT)) or histopathological correlates and the results were within PSL historical control range values [17].

Anemia is characterized as a reduction in the amount of RBCs and/or HGB present in circulating blood and classified morphologically or etiologically [18]. For a morphological classification of anemia, which is driven by the size of RBCs and concentrations of HGB, changes in mean corpuscular volume (MCV), mean corpuscular hemoglobin concentration (MCHC), and MCH can be informative [18]. When decreases in RBCs occur, changes in MCHC, MCV, and RDW have been used as supportive data in a “weight-of-evidence approach” to support a diagnosis of anemia or other potential cause(s) for the decrease in RBCs [19]. The MCH is a calculation of the average concentration of HGB in the blood divided by the total RBC [18]. The RDW is an evaluation of anisocytosis, variation in the size of circulating RBCs [18], with a high RDW indicative of variation in the size of RBCs and a low RDW indicative of uniform size among RBCs. The MCV is a measure of the average volume of RBCs (*i.e.*, the volume of total RBC divided by total RBC). There is no relationship between anisocytosis and MCV, as variation in the size of RBCs may occur for all values of MCV [18]. Tomita et al. [20] reported that a 2-year dietary treatment with *p,p*’-DDT resulted in microcytic anemia in rats as supported by significant decreases in HCT, HGB, RBC, MCV, and MCH after 12-weeks of treatment and significant decreases in HCT, HGB, MCV, and MCH after 26, 52, and 78 weeks of treatment. As shown in Table 2 (male) and Table 3 (female), there were no statistically significant changes reported in rats in either RBC or HGB at any of the doses tested, as well as in MCV or MCHC, supporting that the changes in MCH and RDW alone are not indicative of anemia or of toxicological significance in this study.

To further support that the statistically significant MCH and RDW changes are not of toxicological concern, the values were compared against the historical control range values for PSL. The statistically significant increase in the MCH for Group 4 male rats of 18.2 ± 0.7 pg was within the historical control range values of 15.3–21.5 pg and equivalent to the historical control mean of 18.2 pg (*N *= 502). The statistically significant decrease in RDW for Group 4 males of 11.9 ± 0.5% was within the historical control range values of 11.1–35.8% (mean of 13.3%; *N *= 502). Similarly, in the Group 3 and Group 4 females, the respective statistically significant decreases in RDW of 11.3 ± 0.4% and 11.4 ± 0.5% were within the historical control range values of 10.1–13.3% (mean of 11.6%; *N *= 498).

The absolute spleen weight, spleen-to-body weight ratio, and spleen-to-brain weight ratio were statistically significantly decreased in Group 4 female rats only. According to the board-certified clinical pathologist, the changes in spleen weight, spleen-to-body weight ratio, and spleen-to-brain weight ratio were not considered to be adverse because they did not have a corresponding statistically significant change in clinical pathology values or a histopathological or clinical sign correlate [17]. Therefore, these changes were not considered toxicologically significant.

In Group 2 and Group 4 male rats, a statistically significant decrease in ANEU was recorded. This change was not observed in female rats in any of the dose groups. The total blood cell counts in the Group 2 and Group 4 males were unchanged and there were no histopathological effects or a dose-response relationship observed. Further, the ANEU result of 2.17 × 10^−3^ ± 0.82 × 10^−3^/μL (Group 2 male) and 1.58 × 10^-3^  ± 0.35 × 10^−3^/μL (Group 4 male) were within the laboratory’s historical control range values of 0.53 × 10^−3^ to 9.39 × 10^−3^/μL (mean of 2.15 × 10^−3^/μL; *N* = 502). For these reasons, the decrease in ANEU was not considered to be of toxicological concern.

Absolute kidney weights for Group 3 and Group 4 males and kidney-to-brain weight ratios in Group 4 males were slightly increased. However, in the absence of kidney weight changes in females and histopathological findings in the kidney, the changes in absolute kidney weights in Group 3 and Group 4 males and kidney-to-brain weight ratios in Group 4 males were not considered of toxicological significance. An incidental decrease in liver-to-body weight ratio was also noted in Group 3 females, which was not considered to be of toxicological relevance in the absence of histopathological changes. Based on the toxicological data from the 90-day dietary study in male and female rats, a NOAEL of 150,000 ppm KALGAE™ or a dietary intake of 7895.2 and 9708.09 mg KALGAE™/kg bw/day in male and female rats, respectively, was established.

## 7 Conclusions

In conclusion, the lack of adverse effects in male and female rats in the 90-day dietary toxicity study, negative results in the *in vitro* bacterial reverse mutation assay, and negative results in the *in vivo* mammalian erythrocyte micronucleus test support the safe use of KALGAE™ as an ingredient in foods. From the 90-day dietary toxicity study in rats, the NOAEL for KALGAE™ is 7895.2 mg/kg bw/day in male rats and 9708.09 mg/kg bw/day in female rats, which was the highest dose tested.

## Conflict of interest

Ms. Brickel and Dr. Matulka report receiving a salary from Burdock Group, a consulting firm engaged by and who received payment from ZIVO Biosciences, Inc. (the study sponsor; Keego Harbor, MI), prior to and during the duration of the submitted work. Dr. Steffek reports personal fees from ZIVO Biosciences, during the conduct of the study and outside the submitted work. In addition, Dr. Steffek has patents 62/295,976, 62/457,566, and 107104744 pending.

## Funding

This work was supported by ZIVO Biosciences, Inc. 2804 Orchard Lake Road, Suite 202 Keego Harbor, MI 48320, USA.
